# The Unusual Resistance of Avian Defensin AvBD7 to Proteolytic Enzymes Preserves Its Antibacterial Activity

**DOI:** 10.1371/journal.pone.0161573

**Published:** 2016-08-25

**Authors:** Geoffrey Bailleul, Amanda Kravtzoff, Alix Joulin-Giet, Fabien Lecaille, Valérie Labas, Hervé Meudal, Karine Loth, Ana-Paula Teixeira-Gomes, Florence B. Gilbert, Laurent Coquet, Thierry Jouenne, Dieter Brömme, Catherine Schouler, Céline Landon, Gilles Lalmanach, Anne-Christine Lalmanach

**Affiliations:** 1 ISP, INRA, Université François Rabelais de Tours, UMR 1282, Nouzilly, France; 2 CEPR, INSERM, Université François Rabelais, UMR 1100, Tours, France; 3 PRC, INRA, Université François Rabelais de Tours, UMR 85, UMR CNRS 7247, Plate-forme d'Analyse Intégrative des Biomolécules, Laboratoire de Spectrométrie de Masse, Nouzilly, France; 4 CBM, CNRS, UPR 4301, Orléans, France; 5 Collegium Sciences et Techniques, Université d’Orléans, Orléans, France; 6 Plate-forme de Protéomique "PISSARO" de l'IRIB, Université de Rouen, CNRS, UMR 6270, Mont-Saint Aignan, France; 7 Department of Oral Biological and Medical Sciences, University of British Columbia, Vancouver, British Columbia, Canada; Centre National de la Recherche Scientifique, FRANCE

## Abstract

Defensins are frontline peptides of mucosal immunity in the animal kingdom, including birds. Their resistance to proteolysis and their ensuing ability to maintain antimicrobial potential remains questionable and was therefore investigated. We have shown by bottom-up mass spectrometry analysis of protein extracts that both avian beta-defensins AvBD2 and AvBD7 were ubiquitously distributed along the chicken gut. Cathepsin B was found by immunoblotting in jejunum, ileum, caecum, and caecal tonsils, while cathepsins K, L, and S were merely identified in caecal tonsils. Hydrolysis product of AvBD2 and AvBD7 incubated with a panel of proteases was analysed by RP-HPLC, mass spectrometry and antimicrobial assays. AvBD2 and AvBD7 were resistant to serine proteases and to cathepsins D and H. Conversely cysteine cathepsins B, K, L, and S degraded AvBD2 and abolished its antibacterial activity. Only cathepsin K cleaved AvBD7 and released Ile4-AvBD7, a N-terminal truncated natural peptidoform of AvBD7 that displayed antibacterial activity. Besides the 3-stranded antiparallel beta-sheet typical of beta-defensins, structural analysis of AvBD7 by two-dimensional NMR spectroscopy highlighted the restricted accessibility of the C-terminus embedded by the N-terminal region and gave a formal evidence of a salt bridge (Asp9-Arg12) that could account for proteolysis resistance. The differential susceptibility of avian defensins to proteolysis opens intriguing questions about a distinctive role in the mucosal immunity against pathogen invasion.

## Introduction

The intestinal tract is constantly exposed to a complex community of microorganisms that includes commensal bacteria but sometimes invasive pathogens that the epithelial interface has to fight. In this battle, defensins play an important role in mucosal innate immunity by displaying antimicrobial activity towards pathogens, in wound repair capacity and in inflammation [1]. They constitute the largest family of cationic antimicrobial peptides present throughout the animal kingdom, and must be constantly ready to act in their host. Among birds, chicken possess a repertoire of 14 avian β-defensins (AvBDs) but no α-defensins, which are restricted to mammals, or θ-defensins, restricted to primates [2]. These chicken defensins are characterized by a typical three-stranded β-sheet structure stabilized by three disulfide bridges between six highly conserved cysteine residues as determined for AvBD2 [3], that constitute the hallmark of all β-defensins during evolution from birds to mammals. Two of them, AvBD1 and AvBD2 formerly known as gallinacin 1 and gallinacin 2, have been isolated from granules of heterophils (avian polynuclear neutrophils) [4,5]. They can be purified with AvBD7 from the bone marrow and display a large antimicrobial spectrum [6]. Heterophils can infiltrate the intestinal tissue of chicken during infection such as *Salmonella* colonization of the caecum, but are barely present at homeostasis [7,8]. However, gene expression of AvBD1 and AvBD2 has been shown in chicken intestinal epithelial cells [9] and more generally in small and large intestine including caecum [2]. These defensins have been associated to the phenotype of resistance to *Salmonella* carriage in the caecum [10]. By contrast, little is known about the proteases that are present in the chicken intestinal tissue in comparison to the well described mammalian intestinal proteases including serine proteases (neutrophil elastase, trypsin, chymotrypsin), aspartyl cathepsin D (Cat D), and cysteine cathepsins [11]. One study reports the proteolytic activity in the chicken intestine, endorsed by cathepsins [12]. Cathepsins have been associated in mammals with inflammatory processes and/or in tissue remodeling. Their functions are determined by some structural motifs conserved over millions of years after the evolutionary trails have diverged, giving multiple evolutionary groups of cysteine cathepsins [13]. It has been proposed that cysteine cathepsins including Cat S might be involved in pathological inflammatory processes such as colitis [14]. Moreover, cysteine cathepsins can impair activities of antimicrobial peptides under other pathological conditions in mucosal tissues [15–17].

This raises the question of the susceptibility of avian defensins to proteolytic degradation by intestinal proteases. While the high resistance of defensins toward proteolysis could be expected, the structural and functional data supporting this hypothesis remain elusive. The main objective of the present study was therefore to analyze the sensitivity of major intestinal AvBDs toward proteolytic degradation and to determine the ability of truncated AvBDs to retain antibacterial properties, and thus maintain host defense capabilities.

## Materials and Methods

### Ethics statement

White Leghorn chickens, histocompatible for the B13 haplotype (GB1 Athens chicken line), were hatched and raised free of specific pathogens at INRA animal facility (Platform for Experimental Infectiology, PFIE, INRA Val de Loire, Nouzilly, France) until 10 weeks of age, in compliance with French and European guidelines for the accommodation and care of animals used for scientific purposes (European Union Directive 2010/63/EU) and under authorization and supervision of official veterinary services (agreement number C-37-175-3 delivered to the PFIE animal facility by the veterinary service of the Departement d’Indre et Loire, France). In order to collect tissues post-mortem, ten chickens were sacrificed at ten weeks of age by anaesthetic overdose (intravenous dose of 50 mg/kg of body weight) of sodium pentobarbital (Merial, France), in compliance with European Union Directive 2010/63/EU for animal killing. Chicken sacrifices were performed by one of the authors and by an animal technician of the PFIE animal facility (INRA Val de Loire), both licensed persons according to the European Union Directive 2010/63/EU. The procedure was performed in strict compliance with legal dispositions applicable in France, mentioning animal euthanasia with only purpose of organ or tissue use is not considered as an experimental procedure and thus not under obligation of submission to ethics committee for approval (Ordinance 2013–118, article R.214-89, published in the Journal Officiel de la République Française # 0032 of the 7^th^ of February 2013, pp 2199).

### Protein extraction from chicken tissues

Bone marrow was collected from femur and tibia of ten euthanatized White Leghorn chickens (see Ethics statement above) as previously described [6], for AvBDs preparation. Approximately 400 mg of various intestinal segments (jejunum, ileum, caecum, and caecal tonsils) were collected and homogenized with Ultra-Turrax^®^ (IKA-Werke) in a conical 10 mL tube (Sarstedt, Nümbrecht, Germany) containing 4 mL of lysis buffer. The lysis buffer contained 50 mM Tris-Hydrochloric acid (Tris-HCl), 1 mM ethylenediaminetetraacetic acid, 0.1% Tween 20, and 1× Halt EDTA-free protease inhibitor cocktail tablets (Roche Diagnostics, Meylan, France) with a final volume of 50 mL of PBS buffer, pH 7.4 (Gibco, Thermo Fisher Scientific, Saint Aubin, France). Tubes were placed at 4°C during 5 min and then centrifuged at 11,200 × g for 10 min at 4°C. The supernatants were collected and stored at 4°C. Protein concentrations were measured using Quick Start^™^ Bradford Protein Assay (Biorad, Marnes-la-Coquette, France) according to the manufacturer’s recommendations.

### SDS PAGE and immunoblotting of intestinal protein extracts

Total proteins (10 μg) were diluted in Laemmli buffer, heated at 90°C for 5 min and then subjected to electrophoresis under reducing conditions (12% SDS PAGE) [18]. Prestained molecular mass marker (Kaleidoscope Prestained Standards, Biorad) was used. Proteins were further transferred onto a nitrocellulose membrane (Hybond ECL, GE Healthcare Life Sciences, Velizy-Villacoublay, France). The membrane was saturated with 5% BSA in PBS, 0.5% Tween 20 (PBS-T) for 1 h. After three washes of 5 min in PBS-T, the membrane was incubated overnight at 4°C under agitation with a goat anti-human cathepsin (Cat) B antibody (R&D Systems, Lille, France) diluted 1:1,000 in PBS-T containing 5% low fat powdered milk. After 3 washes for 5 min in PBS-T buffer, the membrane was incubated for 1 h with a rabbit anti-goat antibody conjugated to horseradish peroxidase (Nordic-MUbio, Susteren, Netherland) at the dilution of 1: 1500 in PBS-T containing 5% low fat powdered milk. After 3 washes for 5 min in PBS-T, proteins were visualized by chemiluminescence (SuperSignal West Pico/SuperSignal West Femto, Thermo Fisher Scientific, Saint Aubin, France), according to the manufacturer’s instructions. All incubations were performed at 4°C. The same protocol was repeated with goat anti-human cathepsins L and S (R&D Systems). Alternatively, western blotting was performed using a mouse anti-human cathepsin K antibody at the dilution of 1:1,000 (Calbiochem, Merck Millipore, Molsheim, France) and a rabbit anti-mouse antibody-horseradish peroxidase conjugate as secondary antibody (Nordic-MUbio).

### Bottom-up proteomic approach to identify proteins from intestinal tissues

The proteins from each gel slice were digested using trypsin as previously described [19]. The extracted peptides were analyzed by on-line nanoflow liquid chromatography-tandem mass spectrometry (nanoLC-MS/MS) using a dual linear ion trap Fourier Transform Mass Spectrometer (FTMS) LTQ Orbitrap Velos (Thermo Fisher Scientific) coupled to an Ultimate^®^ 3000 RSLC Ultra High Pressure Liquid Chromatographer (Dionex, Thermo Fisher Scientific). Samples were loaded on an LCPackings trap column (Acclaim PepMap 100 C18, 100 mm i.d6 2 cm long, 3 μm particles) and desalted for 10 min at 5 mL/min with 4% solvent B. Mobile phases consisted of solvent A (0.1% formic acid, 97.9% water, 2% acetonitrile, v/v/v) and solvent B (0.1% formic acid, 15.9% water, 84% acetonitrile, v/v/v). Separation was conducted using a LCPackings nano-column (Acclaim PepMap C18, 75 mm i.d6 50 cm long, 3 μm particles) at 300 nl/min by applying gradient consisted of 4 to 55% of solvent B for 90 min. The mass spectrometry analyses were performed in positive ion mode and in data-dependent mode with high resolution (R = 60,000) full scan MS spectra (profile mode) and low-resolution CID-MS/MS (centroid mode). In the scanning range of m/z 300–1800, the 20 most intense peptide ions with charge states of ≥2 were sequentially isolated (isolation width, 2 m/z; 1 microscan) and fragmented by CID with normalized collision energy of 35%. An activation q = 0.25 and activation time of 10 ms were used. Dynamic exclusion was applied during 30 s with a repeat count of 1. Polydimethylcyclosiloxane (m/z, 445.1200025) ions were used as lock mass for internal calibration. All raw data files were converted, processed and confronted to the chordata section of a reference copy of nrNCBI (3326079 sequences, download 01/22/2014), using search parameters as previously described [20].

### Preparation of AvBDs and top-down proteomic analysis

AvBDs were purified from chicken bone marrow according to the procedure previously described [6]. After size-exclusion chromatography of the bone marrow peptide extract, fractions were diluted (v/v) with a mixture of water-formic acid-methanol (v:v:v; 49:1:50) and were directly analyzed by top-down proteomic approach for structural identification of native AvBDs using a dual linear ion trap Fourier Transform Mass Spectrometer (FTMS) LTQ Orbitrap Velos (Thermo Fisher Scientific) as previously described [21]. All analyses were performed using a high-high strategy, meaning that a FTMS spectra using profile mode in the mass range m/z 400–1500, was followed by an FTMS2 spectra obtained by HCD (normalized collision energy between 40–60%). Target resolution was 100,000 for FTMS and FTMS2 analysis. The spectrum shown in this study correspond to the accumulation of scans over approximately 1 min, yet good signal to noise ratios could be obtained within less time. Raw data were integrated in ProSight PC software (Thermo Fisher Scientific) and processed by THRASH (signal/noise: 2–3). FTMS data were confronted directly to AvBD sequences using “single protein” search option. Prosight PC was used with monoisotopic precursors, 15 ppm for fragment ions mass tolerance and the delta mass feature deactivated. Post-translational modifications such as disulfide bridges, N-terminal pyroglutamic acid and C-terminal amidation were interpreted using the manual Sequence Gazer mode. Proposed results with a P score of < 0.05 were considered positively identified and structurally characterized with a minimal 5 fragment ions matching. Finally, RP-HPLC purification was performed as previously described [6], from pools of positive fractions for AvBD2 or AvBD7 as determined, and about one milligram of both full-length AvBD2 and AvBD7 was obtained for each preparation.

### Hydrolysis of AvBD2 and AvBD7

Human Cat K was produced as described previously [22]. Human Cat B, Cat D, Cat H, Cat L and Cat S were purchased from Calbiochem (VWR International, Fontenay-sous-Bois, France). Trypsin and chymotrypsin were purchased from Euromedex (Souffelweyersheim, France). Human neutrophil elastase (HNE) was supplied by BioCentrum (Krakow, Poland). Active site concentrations of proteases were determined as previously described [23]. AvBD2 and AvBD7 (12.5 μM) were incubated in the presence of 125 nM of trypsin, chymotrypsin, HNE, Cat B, Cat D, Cat H, Cat K, Cat L or Cat S. Assays for cysteine cathepsins (B, H, K, L and S) were carried in 0.1 M sodium acetate buffer, pH 5.5 containing 2 mM DTT and 0.01% Brij35 for 4 h at 30°C. The reaction was stopped by adding 4 μM of E-64 (L-3-carboxy-*trans*-2, 3-epoxypropionyl-leucylamido-(4-guanidino) butane, Sigma-Aldrich, Saint-Quentin-Fallavier, France). Alternatively, activity buffers were 0.1 M Tris/HCl buffer, pH 7.8, 20 mM CaCl_2_ for chymotrypsin, 0.1 M Tris/HCl buffer, pH 8.0, 50 mM CaCl_2_, 100 mM NaCl for trypsin, 0.05 M HEPES buffer, pH 7.4, NP40 0.05% 150 mM NaCl for HNE, and 0.1 M sodium citrate buffer, pH 3.5, 200 mM NaCl for Cat D, respectively. Following 4-hour incubation, serine proteases were inactivated by 4-(2-Aminoethyl-benzenesulfonyl fluoride hydrochloride (10 μM) (Pefabloc, Sigma-Aldrich) while Cat D was inhibited by pepstatin A (10 μM) (Sigma-Aldrich), before removal of 4 μL of the reaction mixture. Incubation products were analyzed by reverse-phase high-performance liquid chromatography (RP-HLC).

### Analysis of cleavage products of defensins

Each reaction mixture was submitted to RP-HPLC (C-18 Lichrocart 55–2 Purospher Star column) using a linear 0–90% water/acetonitrile gradient in the presence of 0.1% TFA, at a flow rate of 0.5 mL/min. Chromatograms were analyzed using the ChromQuest software (Thermo Fisher Scientific). Major peaks were collected, lyophilized, and resuspended in H_2_O.

The major peak produced by the AvBD7 / Cat K reaction was collected and concentrated. The sample was solubilized with 10 μL acetonitrile/trifluoroacetic acid/water (30/1/69 v/v) and 0.5 μL was deposited on MALDI plate with equal volume of α-Cyano-4-HydroxyCinnamic Acid (CHCA) matrix solution at 5 mg/mL in acetonitrile/trifluoroacetic acid/water (50/0.1/49.9 v/v). After drying of the droplet in ambient air, the sample was inspected by MALDI-TOF mass spectrometry using a MALDI-TOF-TOF Ultraflex (Bruker Daltonics, Wissembourg, France) in reflector mode with a pulsed-ion extraction (PIE) delay of 180 ns and an accelerating voltage in the ion source of 25 kV. Calibration was performed with peptides of known molecular mass (1−2.5 kDa range): Angiotensin II, Angiotensin I, Neurotensin, ACTH clip (1–17) and ACTH clip (18–39). The accuracy of mass determinations was ±0.02%. The identification of AvBD7 cleavage sites was determined by comparing the experimentally measured peptide masses to the theoretical peptides masses from AvBD sequences with the FindPept Tool accessible to SIB Bioinformatics Resource Portal (http://web.expasy.org/cgibin/%22ndpept/%22ndpept_form.pl).

The major peak produced by the AvBD7 / Cat K reaction was also analyzed by N-terminal sequencing. The protein of interest was loaded onto a precycled *Biobren Plus-*coated glass filter. The N-terminal sequence was determined by introducing the filter into an Applied Biosystems 494 automated protein sequencer (Life Technologies SAS, Villebon sur Yvette, France) and runs of Edman degradation were carried out. The residues obtained were matched to the expected sequence of defensin.

### Antibacterial properties of defensins and cleavage products

The antibacterial activity of purified AvBD2 and AvBD7 and of each enzyme-AvBD reaction mixture was measured by radial diffusion assay [24] as described previously [6], allowing the determination of the minimal inhibitory concentration (MIC) against *Staphylococcus aureus* ATCC 29740 and against *Escherichia coli* ATCC 25922 as representative Gram-positive and Gram-negative strains, respectively. The reaction mixture without defensin added was used as negative control and the defensin alone was used as positive control. For AvBD7 and its major degradation product, MIC was determined towards three Gram-negative and three Gram-positive bacterial strains, respectively, including *Pseudomonas aeruginosa* ATCC 25010, *Escherichia coli* ATCC 25922, *Salmonella enterica* serovar Typhimurium LT2 ATCC 700720, *Listeria monocytogenes* strain EGD, *Staphylococcus aureus* ATCC 29740, and *Streptococcus salivarius* JIM 8780. The strain *Salmonella enterica* serovar Typhimurium LT2 was kindly provided by Dr Benoît Doublet (INRA, UMR1282 ISP, Nouzilly, France). The strain *Streptococcus salivarius* was a clinical strain isolated from blood culture, kindly provided by Dr Christine Delorme (INRA, UMR1319 MICALIS, Jouy en Josas, France).

### Statistical analysis

A Mann-Whitney non-parametric U test was used to compare the minimal inhibitory concentration of each defensin before and after incubation with cathepsins.

### Analysis of AvBD7 structure

2D ^1^H TOCSY (80ms) and NOESY (160 ms) and a sofast-HMQC [25] (^15^N natural abundance, 704 scans) experiments were performed at 298K on a 0.23 mM solution of AvBD7 in H_2_O:D_2_O (9:1 ratio) at pH 4.5 on an Avance III HD BRUKER 950 MHz spectrometer equipped with a cryoprobe. Identical sets of TOCSY/NOESY experiments were recorded at 288K and 308K on an Avance III HD BRUKER 700 MHz spectrometer equipped with a cryoprobe, to resolve assignment ambiguities due to spin overlaps. After lyophilization, the same sample was dissolved in D_2_O to record the same homonuclear experiments and a ^13^C-HSQC (natural abundance, 1184 scans, 700 MHz spectrometer with a cryoprobe). ^1^H chemical shifts were referenced to the water signal (4.87 ppm at 288K, 4.77 ppm at 298K and 4.68 ppm at 308K). NMR data were processed using Bruker's Topspin 3.2^™^ and analyzed with CCPNMR (version 2.2.2) [26].

### Structure calculation

Structures were calculated with NOE derived distances, hydrogen bonds (in accordance with the observation of a β-sheet typical long distance NOE cross peaks network—H^N^/H^N^, H^N^/H^α^, H^α^/H^α^) and backbone dihedral angle restraints (determined with the DANGLE program [27]) using CNS [28,29] through the automatic assignment software ARIA2 (version 2.3) [30]. The pyroglutamic acid residue (pQ) located at the N-terminal position of the AvBD7 sequence is considered as a nonstandard residue in CNS. Topology libraries (topalldg5.3.pro and topalldg5.3.pep) were modified as described in the ARIA 2.3 tutorials. Covalent bonds were added between sulfur atoms involved in each bridge (Cys11-Cys40, Cys18-Cys33 and Cys23-Cys41) by homology with other β-defensins and in accordance with intra-cysteine NOE connectivities [Cys11(H^N^)/Cys40(H^β^), Cys18(H^N^)/Cys33(H^β^), Cys18(H^α^)/Cys33(H^β^), Cys23(H^β^)/Cys41(H^β^), Cys41(H^N^)/Cys23(H^β^), Cys41(H^α^)/Cys23(H^β^)]. The ARIA2 protocol used simulated annealing with torsion angle and Cartesian space dynamics with the default parameters. The iterative process was repeated until the assignment of the NOE cross peaks was complete. The last run used a list of 904 NOE-derived distance restraints and was performed with 1000 structures at each iteration. Finally 200 structures were refined in water, and the 10 best structures were selected on the basis of total energies and restraint violation statistics to represent AvBD7 in solution. The quality of the final set of structures was evaluated using PROCHECK-NMR [31] and PROMOTIF [32] software. The figures were prepared with PYMOL [33]. Electrostatic and hydrophobic areas were calculated at the Connolly surface, by APBS [34] and by Platinum [35] software, respectively. The atomic solvent accessible areas were determined by the NACCESS program [36].

## Results

### Detection of cathepsins and defensins in chicken intestinal tissues

**Fig 1:**
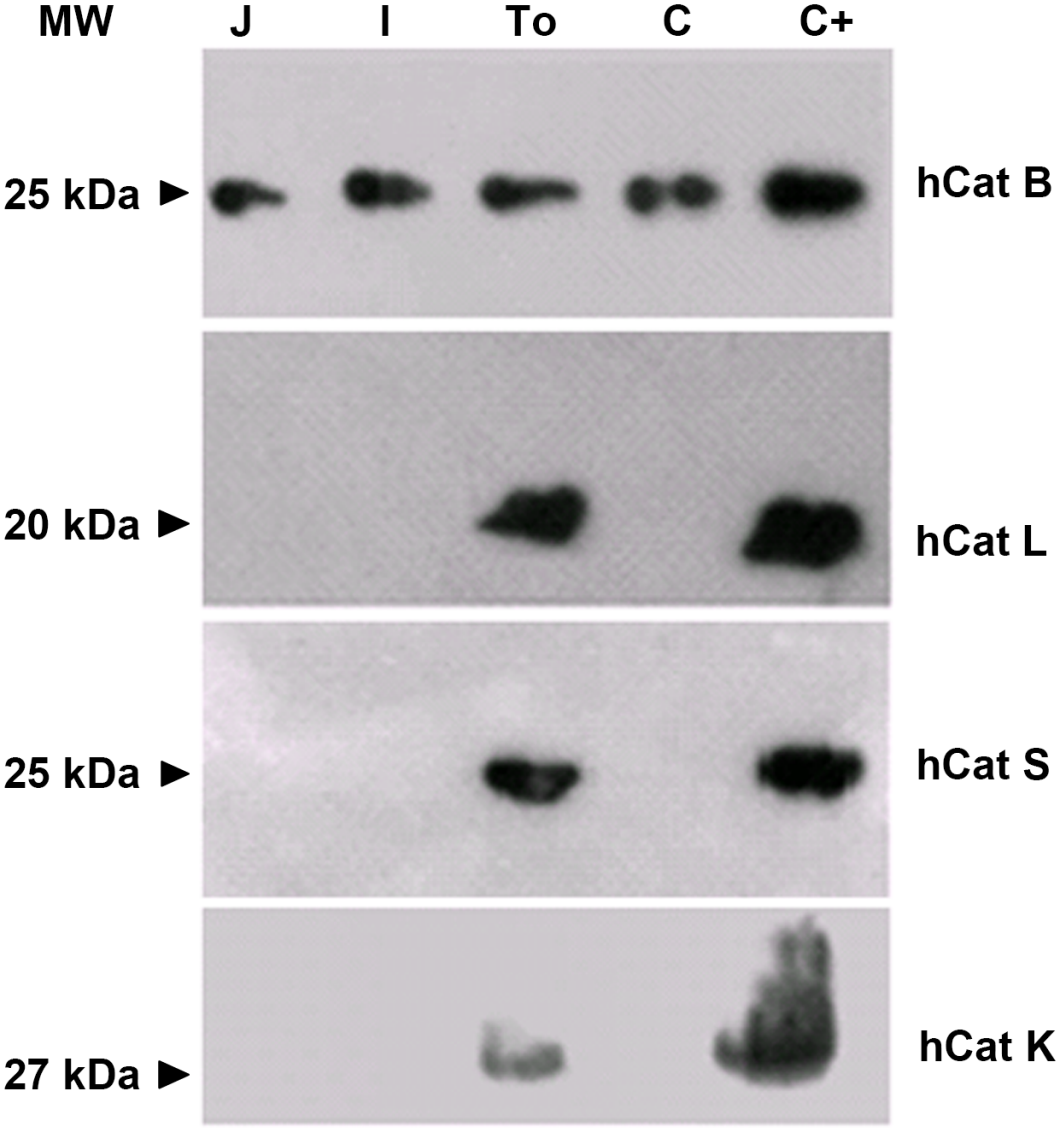
Immunodetection of cathepsins in chicken intestinal segments. Protein extracts from chicken jejunum (J), ileum (I), caecal tonsil (To), and caecum (C), as well as human cathepsins used as positive control (C+), were subjected to SDS-PAGE (12%) before immunoblotting analysis. Each human cathepsin (hCat) is indicated on the right side, with its molecular weight (MW) indicated on the left side.

We initially studied the presence of cysteine cathepsins in sequential segments of chicken intestinal tract (jejunum, ileum, caecum and caecal tonsils) that were previously reported to express AvBD genes [2]. It should be noted that no commercial anti-chicken cathepsin antibodies are currently available. However, anti-human antibodies directed towards Cat B, Cat K, Cat L, and Cat S displayed a substantial cross reactivity with the avian enzymes when tested by western blotting (Fig 1). This cross reactivity is supported by the percentage of identity of 77% for Cat B, 80% for Cat K, 69% for Cat L and 67% for Cat S between human and chicken proteases (determined by NCBI BLAST tool, http://blast.ncbi.nlm.nih.gov/Blast.cgi). Cat B was identified in protein extracts from jejunum, ileum, caecum, and caecal tonsils of birds (Fig 1). Conversely Cat K, Cat L and Cat S were only revealed in caecal tonsils. In parallel, aspartyl Cat D was detected by high-resolution mass spectrometry in ileum, caecum and tonsils, but not in jejunum, while Cat H was uncovered only in caecal tonsils (Table 1). AvBD1 was also detected in intestinal sections, except ileum. The exhaustive list of all the proteins identified in each intestinal segment is provided as supplementary data (see S1 Table in the supplemental material). Both AvBD2 (with a higher relative abundance) and AvBD7 were found in the four intestinal segments, i.e. jejunum, ileum, caecum and tonsils (Table 1). Finally, caecal tonsil is a restricted intestinal site where defensins and cathepsins are expressed and thus may both be encountered.

**Table 1:** Identification of AvBDs and cathepsins in protein extracts from intestinal segments analysed by bottom-up mass spectrometry (nanoLC-MS/MS).

Identified Proteins	Accession Number^a^	Number of Unique Peptides				Protein Identification Probability				EmPAI^c^			
		To^b^	C	I	J	To	C	I	J	To	C	I	J
AvBD2	gi|385251609	4	5	3	4	100%	100%	100%	100%	197.44	259.43	30.79	67.44
AvBD7	gi|304282216	3	3	2	2	100%	100%	100%	100%	4.48	2.62	2.77	1.58
Cathepsin D precursor [*Gallus gallus*]	gi|45384002	3	3	3	0	100%	100%	100%	42%	0.60	0.15	0.48	0
Cathepsin B [*Homo sapiens*]	gi|16307393	1	0	1	0	98%	99%	100%	99%	0.15	0	0.09	0
Cathepsin B precursor [*Gallus gallus*]	gi|46195455	0	0	1	1	0	98%	100%	98%	0	0	0.09	0.22
AvBD1 Precursor	gi|73915343	1	1	0	1	86%	84%	99%	99%	0.85	0.29	0	0.57
AvBD1 Precursor	gi|114053822	1	0	0	1	99%	0	92%	81%	0.88	0	0	0.59
Cathepsin H [*Gallus gallus*]	gi|330376140	1	0	0	0	99%	32%	38%	33%	0.16	0	0	0
Cathepsin L1 [*Homo sapiens*]	gi|148745204	1	0	0	0	100%	27%	0	0	0.15	0	0	0

^a^ NCBInr accession number.

^b^ To, caecal tonsils; C, caecum; I, ileum; J, jejunum.

^c^ Exponentially modified protein abundance index.

### Resistance of AvBDs to proteolysis

**Fig 2:**
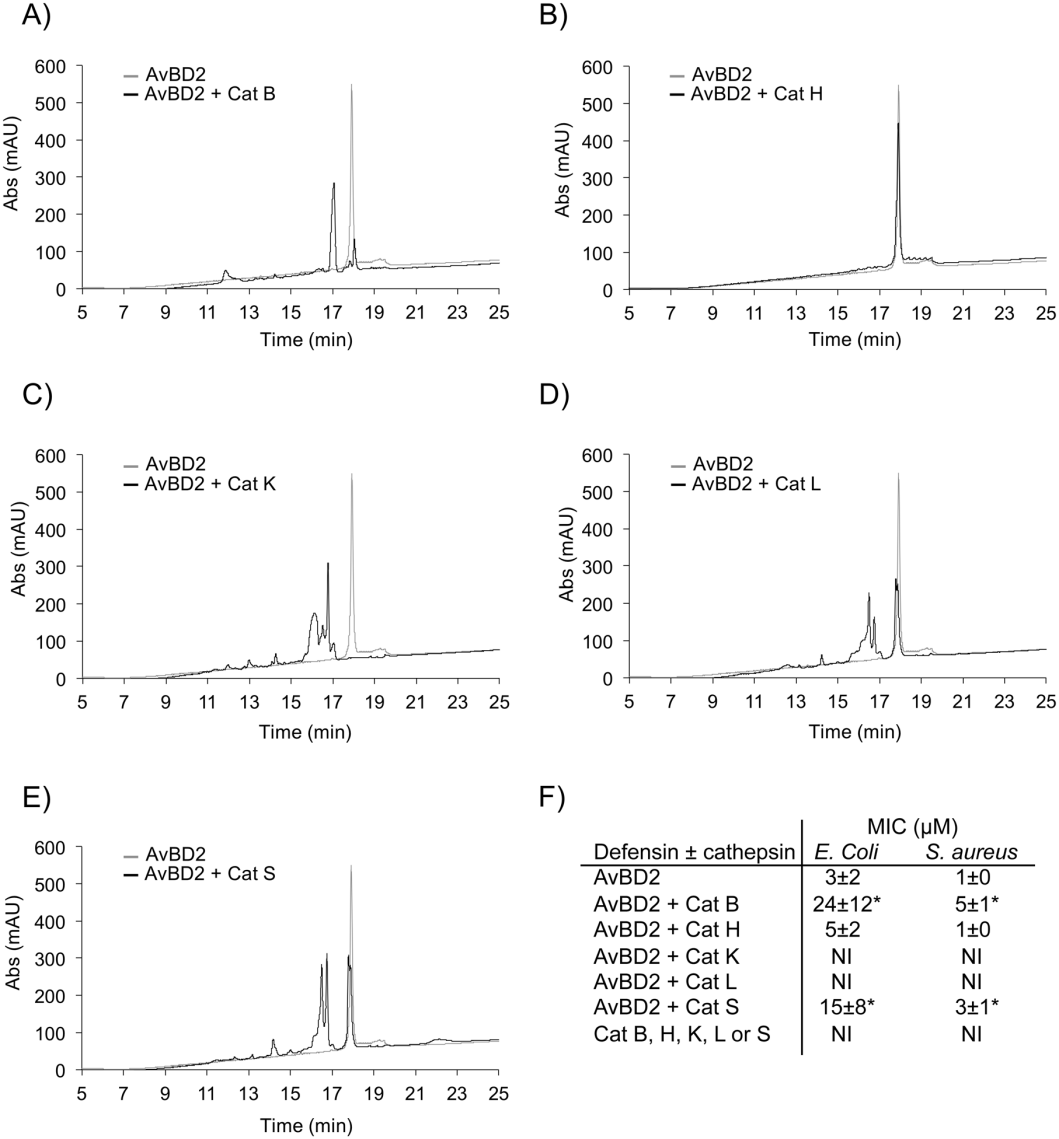
Analysis of AvBD2 degradation products by RP-HPLC (A-E) and by antimicrobial assay (F). AvBD2 was incubated in the presence of Cat B (panel A), Cat H (panel B), Cat K (panel C), Cat L (panel D), and Cat S (panel E) (S:E molar ratio = 100) in 0.1 M sodium acetate buffer, pH 5.5 containing 2 mM DTT and 0.01% Brij35 for 4 h at 30°C (black line). Control experiments used untreated AvBD2 (light grey line). Hydrolysis products were analyzed by RP-HPLC as described in Experimental Procedures. Chromatograms were recorded at 220 nm. In panel F, minimum inhibitory concentrations (MIC) in μM were calculated by radial diffusion assay for each reaction mixture towards *E*. *coli* and *S*. *aureus*, and expressed as mean ± SEM indicated in parenthesis (n = 3). NI: no inhibition detected. Significant differences of MIC values between AvBD2 and its reaction product are indicated with asterisks (*, P< 0.05).

**Fig 3:**
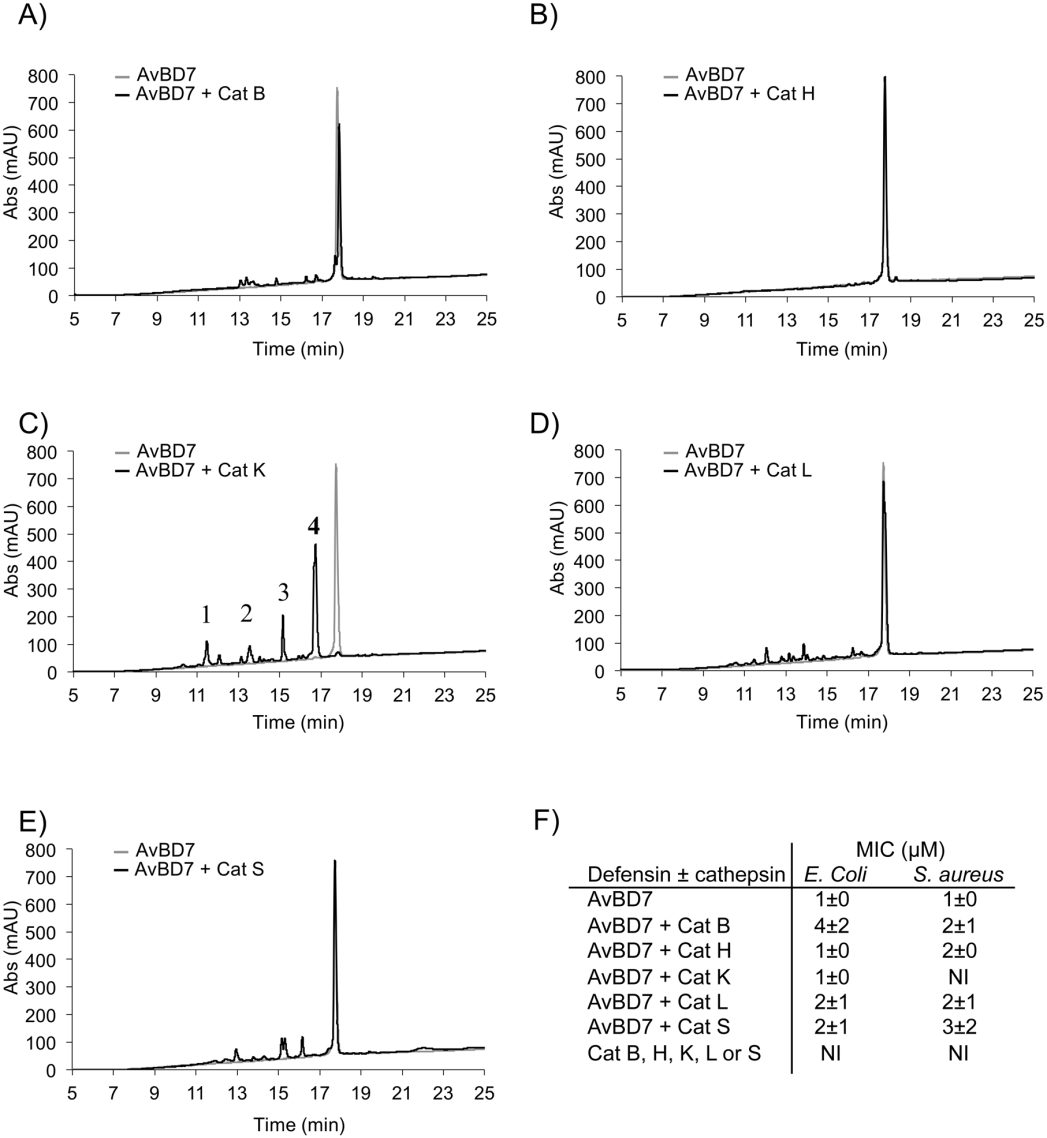
Analysis of AvBD7 degradation products by RP-HPLC (A-E) and by antimicrobial assay (F). AvBD7 was incubated in the presence of Cat B (panel A), Cat H (panel B), Cat K (panel C), Cat L (panel D), and Cat S (panel E) (S:E molar ratio = 100) in 0.1 M sodium acetate buffer, pH 5.5 containing 2 mM DTT and 0.01% Brij35 for 4 hours at 30°C (black line). Control experiments used untreated AvBD7 (light grey line). Hydrolysis products were analyzed by RP-HPLC as described in Experimental Procedures. Chromatograms were recorded at 220 nm. In panel F, minimum inhibitory concentrations (MIC) in μM were calculated by radial diffusion assay for each reaction mixture towards *E*. *coli* and *S*. *aureus*, and expressed as mean ± SEM indicated in parenthesis (n = 3). NI: no inhibition detected.

All forms of AvBDs separated by size-exclusion chromatography from the chicken bone marrow protein extract were analyzed by high-resolution top-down mass spectrometry (see S1 Fig in the supplemental material). Results revealed the presence of peptidoforms of AvBD1, AvBD2, and AvBD7 with variable truncations of their N- and/or C-termini (up to 3 amino acids) as summarized in Table 2, according to the mass of the peptidoforms identified (see S1 Fig in the supplemental material). The full-length forms of AvBD2 and AvBD7 were further purified by RP-HPLC. AvBD2 and AvBD7 were incubated with trypsin, chymotrypsin, and HNE, aspartyl Cat D and cysteine Cat B, Cat H, Cat K, Cat L, Cat S (4 hours; substrate: enzyme molar ratio of 100) respectively. Resulting hydrolysis products were further analyzed by RP-HPLC. Trypsin, chymotrypsin, neutrophil elastase and Cat D did not cleave AvBD2 and AvBD7 (see S2 Fig in the supplemental material). Moreover both defensins were resistant to hydrolysis by Cat H (Figs 2B and 3B). AvBD2 was partially degraded by Cat B, Cat L, and Cat S (Fig 2A, 2D and 2E), leading to the partial loss of antimicrobial activity towards *Escherichia coli* and *Staphylococcus aureus* as attested by the significant increase of its MIC (Fig 2F). Under these experimental conditions, AvBD2 was totally digested by Cat K (Fig 2C), as confirmed by complete loss of antimicrobial activity (Fig 2F). By contrast, AvBD7 was fully resistant to hydrolysis by Cat B, Cat L and Cat S (Fig 3A, 3D and 3E) and retained antimicrobial activity (Fig 3F). Cat K was the only protease that hydrolyzed extensively AvBD7 (Fig 3C), without increasing its MIC towards *E*. *coli* but abolishing its antibacterial effect towards *S*. *aureus* (Fig 3F). Cat K was able to generate a major degradation product (peak 4, Fig 3C) for which antimicrobial activity was further determined towards additional Gram-positive and Gram-negative strains. Of major interest, the AvBD7-derived peptide (i.e. peak 4) displayed similar antibacterial properties to that of uncleaved native AvBD7, except against *Staphylococcus aureus* (Table 3).

**Table 2:** Characterization of AvBDs peptidoforms from bone marrow peptide extract by top-down mass spectrometry.

Peptide name	Sequence	Modifications	Observed Monoisotopic [M+H]+	Theoretical Monoisotopic [M+H] +
AvBD1 full length	GRKSDCFRKSGFCAFLKCPSLTLISGKCSRFYLCCKRIW	3 DB^a^ + C-ter amidation	4501.267	4501.223
AvBD1 peptidoform 1	—KSDCFRKSGFCAFLKCPSLTLISGKCSRFYLCCKRIW	3 DB + C-ter amidation	4288.140	4288.101
AvBD2 full length	LFCKGGSCHFGGCPSHLIKVGSCFGFRSCCKWPWNA	3 DB	3913.738	3913.717
AvBD2 peptidoform 1	LFCKGGSCHFGGCPSHLIKVGSCFGFRSCCKWPWN-	3 DB	3842.709	3842.680
AvBD2 peptidoform 2	LFCKGGSCHFGGCPSHLIKVGSCFGFRSCCKWPW—	3 DB	3728.664	3728.637
AvBD2 peptidoform 3	-FCKGGSCHFGGCPSHLIKVGSCFGFRSCCKWPWN-	3 DB	3729.667	3729.596
AvBD2 peptidoform 4	-FCKGGSCHFGGCPSHLIKVGSCFGFRSCCKWPWNA	3 DB	3800.667	3800.633
AvBD7 full length	QPFIPRPIDTCRLRNGICFPGICRRPYYWIGTCNNGIGSCCARGWRS	3 DB + N-ter pyroglutamic acid	5350.556	5350.513
AvBD7 peptidoform 1	QPFIPRPIDTCRLRNGICFPGICRRPYYWIGTCNNGIGSCCARGWR-	3 DB + N-ter pyroglutamic acid	5263.527	5263.481
AvBD7 peptidoform 2	---IPRPIDTCRLRNGICFPGICRRPYYWIGTCNNGIGSCCARGWRS	3 DB	4995.393	4995.360
AvBD7 peptidoform 3	---IPRPIDTCRLRNGICFPGICRRPYYWIGTCNNGIGSCCARGWR-	3 DB	4908.362	4908.328

^a^ DB: disulfide bridges

**Table 3:** Antimicrobial activities of AvBD7 and of Ile4-AvBD7.

Bacterial strains	MIC (μM)^a^	
	AvBD7	Ile4-AvBD7
***Gram +***		
*Streptococcus salivarius*	0.7 (± 0.2)	1.0 (± 0.5)
*Listeria monocytogenes*	0.7 (± 0.4)	0.2 (± 0.1)
*Staphylococcus aureus*	0.5 (± 0.4)	NI
***Gram -***		
*Escherichia coli*	1.0 (± 0.1)	0.5 (± 0.1)
*Salmonella* Typhimurium	2.6 (± 0.6)	1.4 (± 0.9)
*Pseudomonas aeruginosa*	0.7 (± 0.3)	0.2 (± 0.1)

^a^ Minimum inhibitory concentration (MIC) values were determined by a radial diffusion assay for every bacterial strain. Values are given in μM as means +/- SEM indicated in parenthesis (n = 3). NI: no inhibition detected.

### Structural investigations on AvBD7

**Fig 4:**
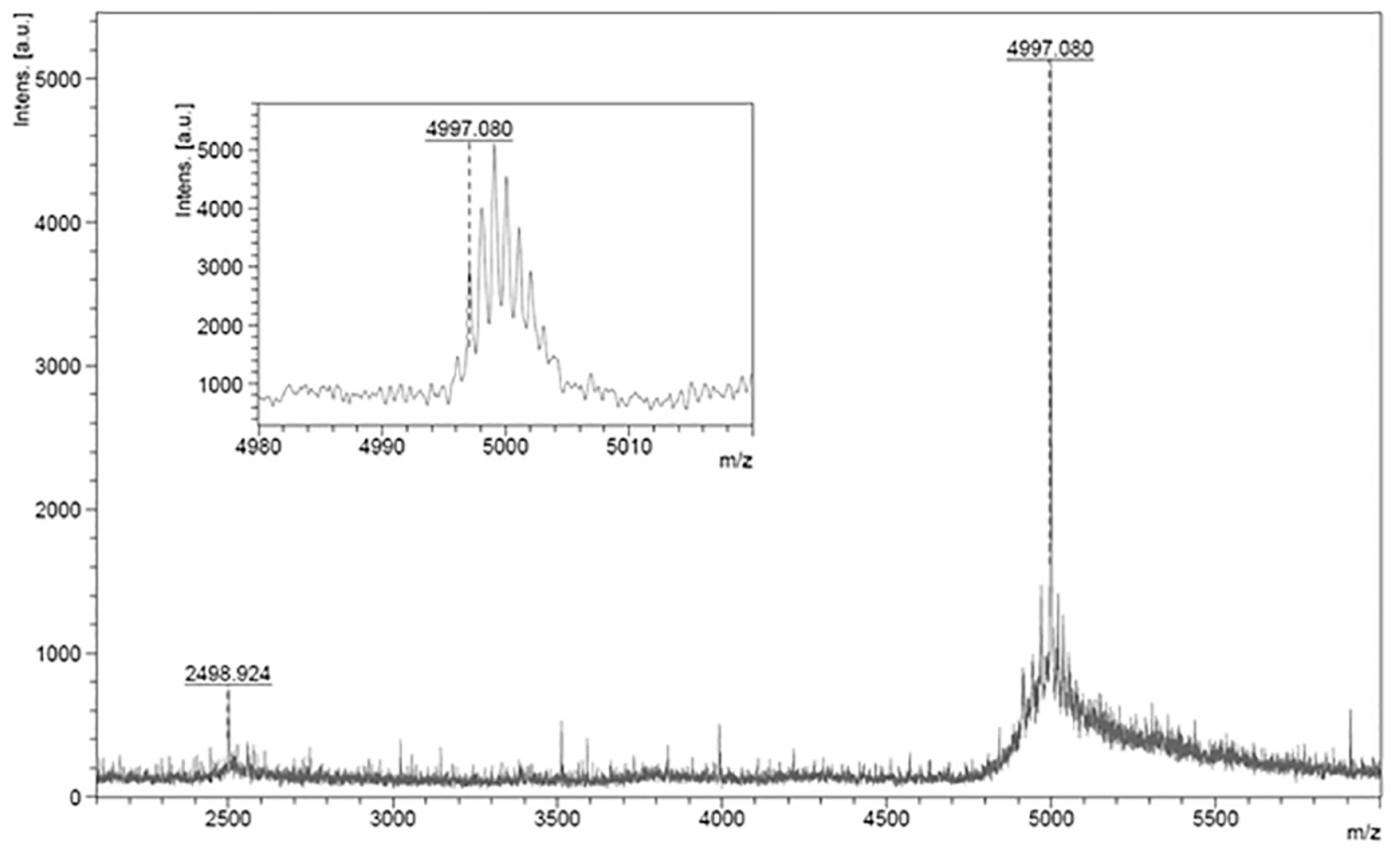
MALDI-TOF mass spectrum of the major hydrolysis product of AvBD7 following incubation with Cat K. The major product corresponds to the N-terminal truncated AvBD7 lacking the first three amino acids (Ile4-AvBD7, i.e. the natural peptidoform 2 of AvBD7 according to Table 2).

The major and active hydrolysis product of AvBD7 by Cat K was further characterized by N-terminal peptide sequencing and by mass spectrometry (Fig 4). Its sequence corresponded to the 3 amino acid truncated form of AvBD7 starting from Ile4 (full-length AvBD7 sequence numbering) and its mass matched to the natural peptidoform 2 of AvBD7 (Table 2). Moreover, taken together these results indicate that the three N-terminal residues are not essential amino acids for AvBD7 antibacterial activity.

The 3D structure of AvBD7 was determined by NMR spectroscopy. The ^1^H homonuclear and the natural-abundance ^15^N heteronuclear spectra revealed a good dispersion of the amide chemical shifts, indicative of a highly structured peptide. Several minor forms co-existed in solution. However, the analysis of the sets of 2D-TOCSY and NOESY spectra allowed a complete assignment of ^1^H chemical shifts (BRMB entry 34014) of the main form. Natural-abundance heteronuclear NMR spectra allowed 39 of the N^H^, 35 of the C^α^ and 33 of the C^β^ shifts to be assigned along with many side chain carbon chemical shifts (BRMB entry 34014). This helped us to unambiguously assign ^1^H chemical shifts, particularly in crowded regions of the ^1^H TOCSY and NOESY spectra corresponding to side chains. The knowledge of H^N^, H^α^, C^α^ and C^β^ chemical shifts allowed us to use the DANGLE program and obtain 70 dihedral angle restraints which supplement the 904 distance restraints derived from NOEs (Table 4). Eight hydrogen bonds and the three disulfide bridges were also introduced in the calculation. Moreover, the Arg25-Pro26 amide bond was set to a *cis* conformation as attested by the typical NOEs: Arg25(H^α^) / Pro26(H^α^) and Arg25(H^N^) / Pro26(H^α^). The use of all these restraints was necessary to reach a good convergence in the calculation. Among the 200 refined structures, 10 structures were selected, in agreement with all the experimental data and the standard covalent geometry, with 95,9% of the residues in the most favored or additionally allowed regions of the Ramachandran diagram (Table 4). This set of structure was considered as representative of the solution structure of AvBD7. Coordinates were deposited in the PDB with entry 5LCS.

**Table 4:** NMR constraints and structural statistics.

**NMR constraints**	
***Distance restraints***	
Total NOE	904
Unambiguous	761
Ambiguous	143
Hydrogen bonds	8
***Dihedral Angle Restraints***	70
***Disulfide bridges***^a^	C11-C40, C18-C33, C23-C41
**Structural Statistics (5LCS.pdb)**	
***Average violations per structure***	
NOEs ≥0.3 Å	0
Hydrogen bonds ≥0.5 Å	0
Dihedrals ≥15°	0
Dihedrals ≥10°	2.1
Average pairwise rmsd (Å)	0.355
***Ramachandran Analysis***	
Most favored region and allowed region	95.9%
Generously allowed	3.2%
Disallowed	0.9%
***Energies (kcal*.*mol***^***-1***^***)***^b^	
Electrostatic	-1376 ± 41
Van der Walls	-385 ± 8
Total energy	-983 ± 41
Residual NOE energy	23 ± 8

^a^ Introduced as covalent bonds.

^b^ Values are given as mean ± standard deviation (n = 10).

**Fig 5:**
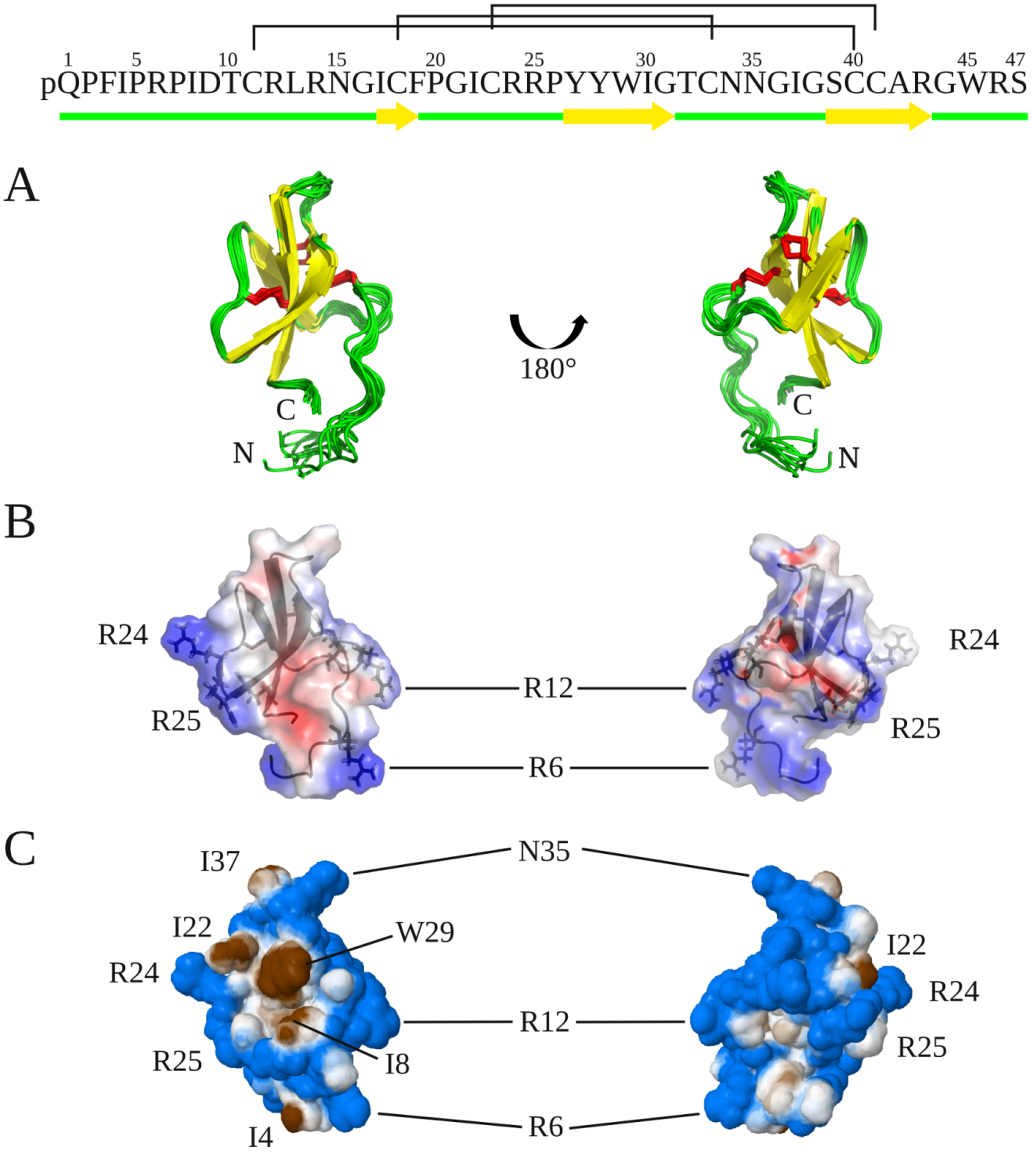
Chicken AvBD7 global fold and surface potentials. Top: AvBD7 sequence. Superimposition of the 10 models representative of chicken AvBD7 solution structure with the three-stranded anti-parallel β-sheet drawn in yellow (A). Electrostatic positive (blue) and negative (red) areas calculated at the Connolly surface by APBS (B). Hydrophobic (brown) and hydrophilic (blue) potential areas calculated at the Connolly surface by Platinum (C).

AvBD7 displayed the typical 3-stranded antiparallel β-sheet of avian defensins, with each strand comprising Cys18-Pro20, Tyr27-Cys33 and Gly38-Arg43 for β1, β2 and β3 respectively as indicated in yellow on Fig 5. In addition, AvBD7 displayed a bulge composed of Cys40, Ile30 and Gly31. The N-terminal region (residues 1–10) appeared mainly unstructured. However, the presence of a salt bridge forming a β-turn between Asp9 and Arg12 and the presence of Cys11-Cys40 disulfide bridge leaded this N-terminal region to embed the C-terminal part (Gly38-Arg46) of AvBD7 (Fig 5A). The proximity of the N-terminal and C-terminal segments is attested by long-range NOEs between Arg46 and Pro5, Ile8, Asp9 and Leu13 protons and between Ser47 and Pro5, Pro7 and Ile8. Electrostatic properties and hydrophobic/hydrophilic potentials were calculated at the Connoly surface. Four positive residues of AvBD7 (Arg6, Arg12, Arg24, Arg25) are well scattered on the surface (Fig 5B), whereas a small hydrophobic patch, formed by Ile8, Ile22 and Trp29, was observed on one face (Fig 5C).

## Discussion

The potential degradation of intestinal AvBD2 and AvBD7 by proteases present and active for the digestive function, i.e. trypsin, chymotrypsin, HNE, aspartyl Cat D, and cysteine Cat B, Cat H, Cat K, Cat L and Cat S, is questionable at homeostasis [12]. We have shown that AvBD2 was unaffected by HNE, trypsin, chymotrypsin, aspartyl Cat D and cysteine Cat H. Conversely AvBD2 was partially cleaved by cysteine Cat B, Cat L, and Cat S and totally degraded by Cat K, resulting in the loss of antimicrobial activity of the hydrolysis products by any of these enzymes. These observations for AvBD2 are in line with a previous report showing the susceptibility of defensins to degradation by cysteine Cat B, Cat L, and Cat S in human [16]. However, since there is not a sufficient collection of annotated substrate cleavage sites (MEROPS database—http://merops.sanger.ac.uk) [37], we were not able to predict the relevance of this finding for AvBD2. Surprisingly, the only protease that cleaved AvBD7 was Cat K, releasing the N-terminally truncated Ile4-AvBD7 (with the corresponding cleavage site: Phe3-Ile4). Cleavage of AvBD7 by Cat K is consistent with its substrate specificity where Pro at P2 and Pro at P2’ are preferred residues [38,39]. In addition, the Cat K-generated Ile4-AvBD7 was found as a natural peptidoform of AvBD7 in chicken bone marrow, the primary ontogenesis site of granulocytes that produce mature avian defensins [6]. Indeed, mammalian Cat K is highly expressed in bone and has been shown to participate in bone remodeling [40]. Taken together these data suggest that the contribution of Cat K in the generation of Ile4-AvBD7 (the natural peptidoform 2 of AvBD7) in chicken bone marrow might be biologically relevant. Interestingly, the generated truncated peptidoform of AvBD7, which lacks the first three N-terminal residues, maintained its antimicrobial activity towards Gram-negative and Gram-positive bacteria, at the notable exception of *S*. *aureus*. This suggests that this N-terminal region has no critical impact on the broad antibacterial spectrum of AvBD7.

**Fig 6:**
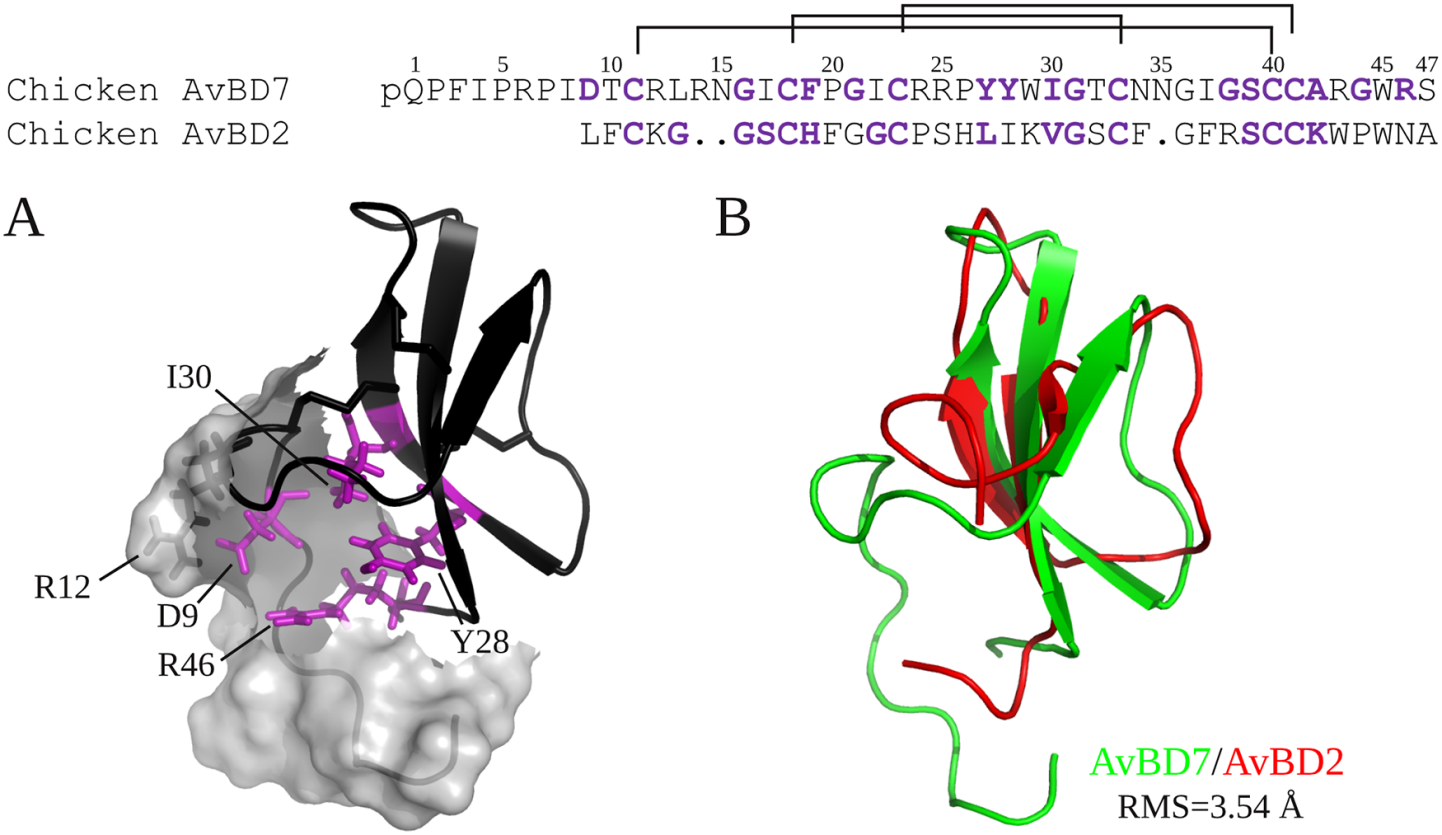
Comparison of AvBD7 and AvBD2 structures. Primary sequence alignment of chicken AvBD7 and AvBD2 (Top). The buried residues are indicated in purple. Cartoon representation of AvBD7 is drawn in black. Surface of the N-term of the protein (1–12) is represented in grey, and residues Asp9, Arg12, Tyr28, Ile30, Arg46 are highlighted in purple (stick representation, buried residues in purple) (A). Superimposition of the global folds of AvBD7 (green) and AvBD2 (red) (B).

The solution structure of AvBD7 appears as compact as other avian or mammalian defensins with the typical 3-stranded antiparallel beta-sheet stabilized by the three conserved cysteine pairings. The bulge, composed of Cys40, Ile30 and Gly31, within the beta-sheet has already been observed in the two other avian β-defensins structures available, namely chicken AvBD2 [3] and King Penguin AvBD103b [41], as well as in mammalian β-defensins. C-terminal residues of AvBD7 are buried, with especially weak accessibility to the solvent and therefore a restricted access to both exo- and endo- proteolytic enzymes. Of peculiar interest Arg46 is fully embedded in the side chains of Tyr27, Tyr28, Ile30 and Trp45 (Fig 6A). Contrary to AvBD2, which only contains two residues before the first cysteine (Fig 6, top), AvBD7 has a long N-terminal tail. Conversely to most vertebrate β-defensin structures containing such a long N-terminal segment upstream the first cysteine residue [41,42] AvBD7 did not show any propensity to form an N-terminal helix. This N-terminal region of AvBD7, overlaying Gly38-Arg46 residues, could play a crucial role in the stability of AvBD7 by conferring an unusual resistance to proteolysis of the buried C-terminus (Fig 6B, [3]). Moreover, Asp9 the unique anionic residue of AvBD7, which is not conserved in other avian defensins, exhibits an unusually low accessibility to the solvent, and forms a salt bridge with Arg12. The presence of this salt bridge that appears as a proper characteristic of AvBD7 (Fig 6A) may also favor resistance of the N-terminus to proteolysis. This can be hypothesized according to the canonical salt bridge described for α-defensins with a protective role against proteolysis [43].

The unaffected antimicrobial activity of AvBD7 after exposure to cathepsins makes it very singular by contrast with the susceptibility of other defense peptides such as LL-37, hBD2 and hBD3 to proteolysis by these enzymes [15,16].

The high degree of conservation of cathepsins throughout evolution is attested by the percentage of identity around 70% between most chicken and human members. This allowed the cross reactivity of anti-human cathepsins antibodies with the respective chicken enzymes and allowed the immuno-detection of Cat B in the various segments of the intestine, from the jejunum, ileum to the caecum, in agreement with the ubiquitous expression of this enzyme along the digestive tract as well as in many other organs [44,45]. In contrast, Cat L, Cat S, Cat K as well as Cat H were only detected in caecal tonsils, a mucosal lymphoid site of birds positioned at the entry of the caecum, which is composed of two appendages of the large intestine containing the most abundant microbiota [46]. The restricted localization of these cathepsins can be explained by their specific involvement in antigen processing [47] in such a sentinel lymphoid tissue dedicated to immune surveillance. In addition, Cat K has been recently associated with both the regulation of inflammation [48] and the processing of chemerin, a chemoattractant molecule with antimicrobial properties within the large intestine [49]. In parallel, the most abundant avian defensins identified by mass spectrometry in chicken caecal tonsils, as well as in other segments of the gut, were AvBD2 and AvBD7. This is consistent with the described production of AvBD2 by enterocytes facing potential invading pathogens [9]. The prominent abundance of AvBD2 in caecal tonsils is also in agreement with former transcriptomic analysis [50]. The probability of the *in vivo* exposure of avian defensins to cathepsins is thus supported by the simultaneous detection of these molecules in the same intestinal segment, i.e. caecal tonsils. This intestinal site is very important for immune surveillance, more especially as it is the site of colonization of pathogenic bacteria such as *Campylobacter* and *Salmonella* [51,52]. However, inflammatory response to infection can be associated with an increase of the proteolytic activity in the intestine, as it has been demonstrated in the context of inflammatory bowel diseases [14,53]. The particularly high level of resistance of AvBD7 to degradation by proteases can be therefore essential to maintain the antimicrobial pressure in the caecum controlling invading pathogens at this portal of entry under proteolytic challenge.

In conclusion, AvBD2, the most abundant avian defensin of the intestinal tissue, was not cleaved by trypsin, chymotrypsin, HNE, Cat D, and Cat H, but was proteolytically inactivated by Cat B, Cat L, Cat S, and Cat K leading to the loss of its antimicrobial activity. Conversely, AvBD7 was exclusively and specifically cleaved by Cat K, releasing the fragment Ile4-AvBD7, a natural peptidoform of AvBD7. Moreover, this peptidoform maintains the antimicrobial activity of full-length AvBD7, demonstrating for the first time the unusually high degree of resistance of this avian defensin to the degradation by gut-resident proteases. The differential susceptibility of two avian defensins to proteolysis opens intriguing questions about a distinctive role in the mucosal immunity against pathogen invasion. It will be particularly interesting to compare their immuno-modulatory activity in the future, in order to evaluate if the extent of the defensins repertoire in animals could be associated to functional diversity.

## Supporting Information

S1 Fig Structural characterization of avian β-defensins 1, 2 and 7 petidoforms by top-down high resolution mass spectrometry (HR-MS). Interpreted nanoESI HR-MS and HR-MS/MS spectra are presented. In each HCD fragmentation spectrum obtained for (A) AvBD2 and (B) AvBD7 and AvBD1, a zoom of the multicharged precursor is presented with the annotated fragmentation spectra with the multicharged b- and y-ions. Identified sequences with the b- and y-type ions observed are indicated in red in the sequence. The three disulfide bridges are taken into account for all forms. C-terminal amidation and N-terminal pyroglutamic acid post-translation modifications are taken into account only for AvBD1 and AvBD7, respectively. Each table resume, for each AvBD, the average precursors selected for MS/MS, the charge of precursor (z), the observed monoisotopic mass [M+H]+, the theoretical monoisotopic mass [M+H]+, the delta mass (Da) between the observed and the theoretical mass, the E-value corresponding to the probability of identification by the ProSight software, the number of b and y ions observed and the total number of ions.
(PDF)

S2 Fig AvBD2 and AvBD7 are resistant to proteolysis by trypsin, chymotrypsin, neutrophil elastase and cathepsin D. (A) AvBD2 (12.5 μM) was incubated in the presence of trypsin, chymotrypsin, neutrophil elastase or cathepsin D during 4 h at 30°C (substrate-enzyme ratio: 100) as described in detail in the experimental section. (B) The same procedure was repeated for AvBD7. Each reaction mixture was submitted to RP-HPLC (Brownlee ODS-032 column, 0–90% water/acetonitrile gradient in the presence of 0.1% TFA, flow rate of 1 mL/min; wavelength: 220 nm). Chromatograms were analyzed by running the ChromoQuest Chromatography Workstation (Thermo Fisher Scientific, Les Ulis, France).
(PDF)

S1 Table Identification of proteins from extracts of intestinal segments. Results are given by computational analysis of bottom-up mass spectrometry (nanoLC-MS/MS) data. For each identified protein, NCBInr accession number is given. Proteins of interest (defensins and cathepsins) are indicated in red.
(XLSX)
