# Supplementary material for: The Unusual Resistance of Avian Defensin AvBD7 to Proteolytic Enzymes Preserves Its Antibacterial Activity
Source: PLoS One. 2016 Aug 25;11(8):e0161573. doi: 10.1371/journal.pone.0161573 (PMC4999073; doi:10.1371/journal.pone.0161573)

A

NanoESI-HRMS spectra for structural characterization of AvBD2 peptidoforms

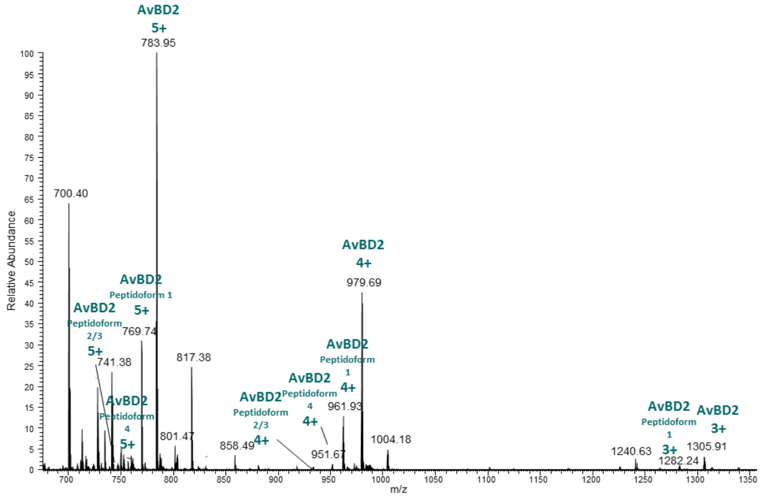

Protein description	m/z precursor (average)	z	Observed monoisotopic mass [M+H]+	Theoretical monoisotopic mass [M+H]+	Delta mass (Da)	Prosight probability E-value	b ions	y ions	Total ions
AvBD 2 full-length	1305.91	3	3913.7380	3913.7178	0.0202	1.14E-04	3	5	8
AvBD 2 peptidoform 1	961.92	4	3842.7092	3842.6807	0.0285	1.E-07	3	7	10
AvBD 2 peptidoform 2	933.41	4	3728.6644	3728.6378	0.0266	9.36E-06	6	4	10
AvBD 2 peptidoform 3	933.41	4	3729.6676	3729.5967	0.0709	9.36E-06	3	6	9
AvBD 2 peptidoform 4	951.16	4	3800.6676	3800.6338	0.0338	4.35E-06	6	4	10

AvBD2 : full-length

LFCKGGSCHFGGCPSHLIKVGSCFGFRSCCKWPWNA

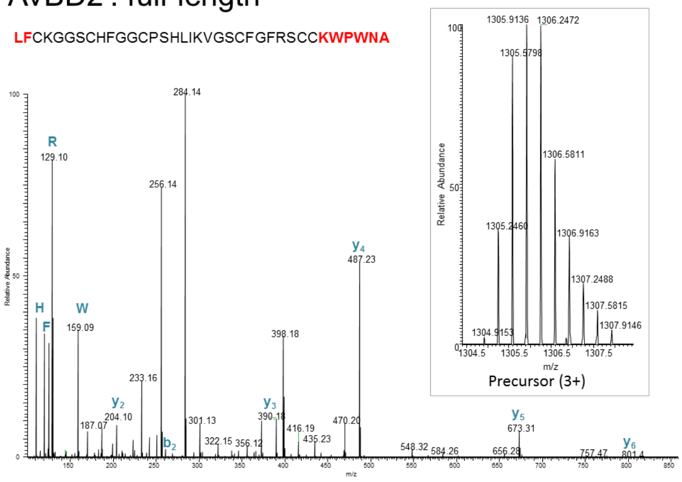

AvBD2 : peptidoform 1

LFCKGGSCHFGGCPSHLIKVGSCFGFRSCCKWPWN

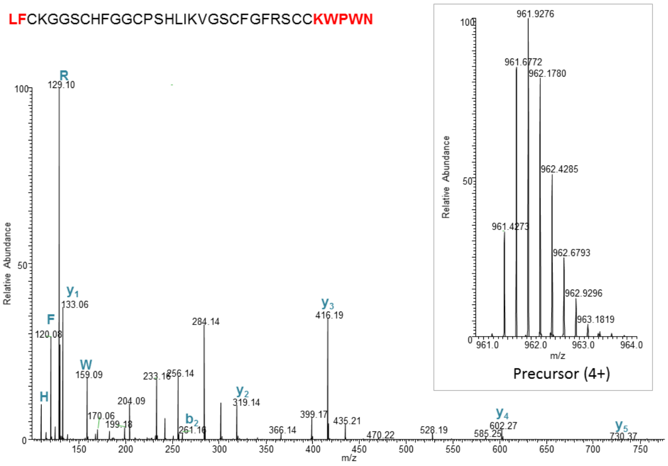

AvBD2 : peptidoform 2 and 3

LFCKGGSCHFGGCPSHLIKVGSCFGFRSCCKWPW

FCKGGSCHFGGCPSHLIKVGSCFGFRSCCKWPWN

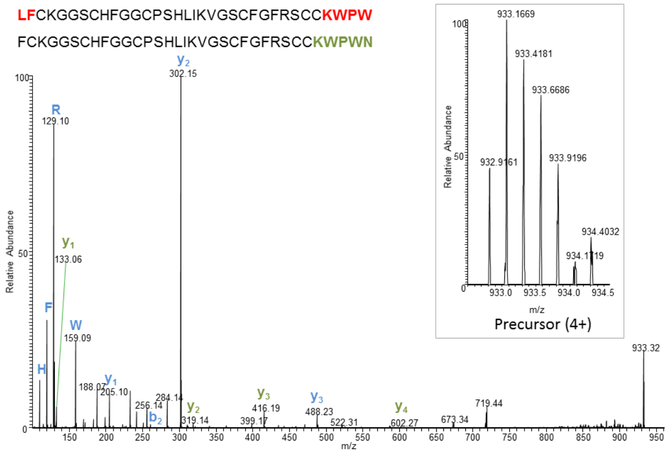

AvBD2 : peptidoform 4

FCKGGSCHFGGCPSHLIKVGSCFGFRSCCKWPWNA

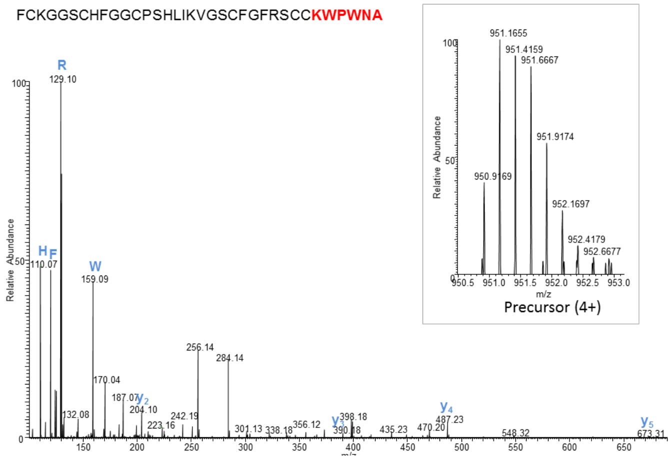

B

NanoESI-HRMS spectra for structural characterization of AvBD1 and AvBD7 peptidoforms

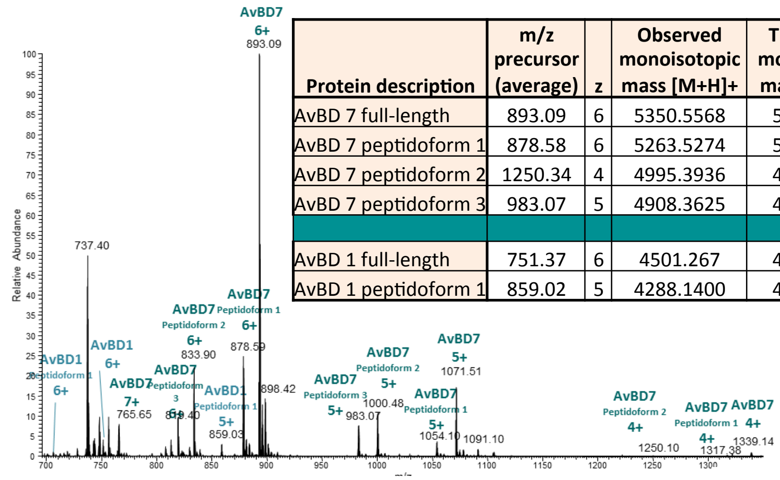

Protein description	m/z precursor (average)	z	Observed monoisotopic mass [M+H]+	Theoretical monoisotopic mass [M+H]+	Delta mass (Da)	Prosight probability E-value	b ions	y ions	Total ions
AvBD 7 full-length	893.09	6	5350.5568	5350.5139	0.0429	1.37E-06	2	13	15
AvBD 7 peptidoform 1	878.58	6	5263.5274	5263.4818	0.0456	2.84E-05	2	8	10
AvBD 7 peptidoform 2	1250.34	4	4995.3936	4995.3607	0.0329	5.08E-06	5	3	8
AvBD 7 peptidoform 3	983.07	5	4908.3625	4908.3287	0.0338	6.68E-08	6	5	11
AvBD 1 full-length	751.37	6	4501.267	4501.2236	0.0434	0.025	5	2	7
AvBD 1 peptidoform 1	859.02	5	4288.1400	4288.1011	0.0389	0.05	3	2	5

AvBD 7 : full-length

PyrQ-PFIPRPIDTCRLRNGICFPGICRRPYYWIGTCNNGIGSCCARGWRS

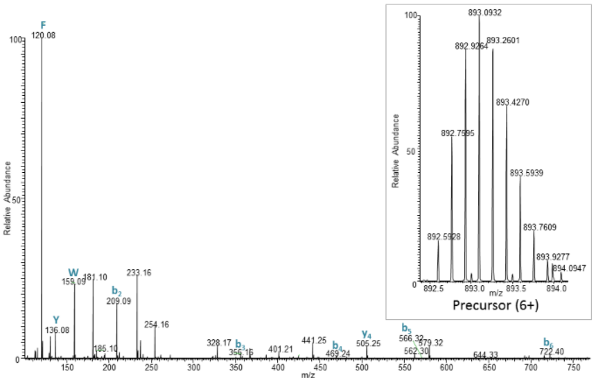

AvBD 7 : peptidoform 1

PyrQ-PFIPRPIDTCRLRNGICFPGICRRPYYWIGTCNNGIGSCCARGWR

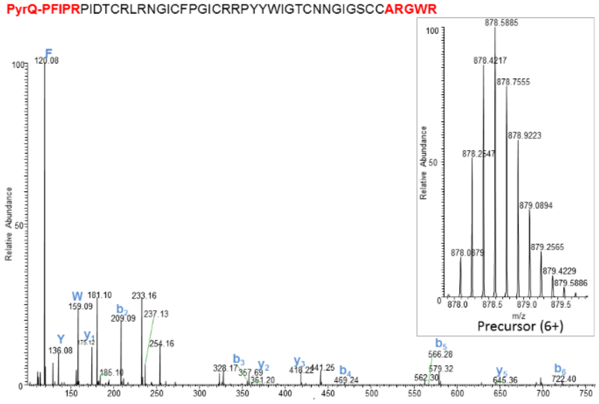

AvBD 7 : peptidoform 2

IPRPIDTCRLRNGICFPGICRRPYYWIGTCNNGIGSCCARGWRS

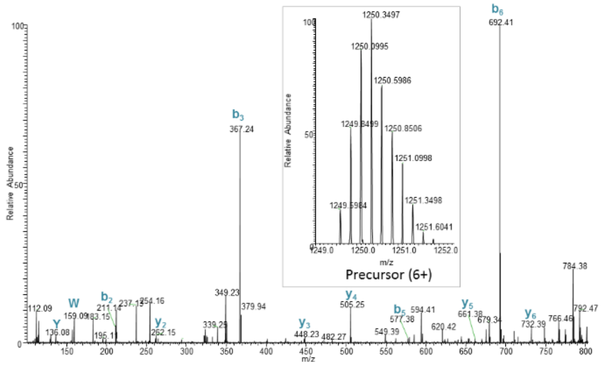

AvBD 7 : peptidoform 3

IPRPIDTCRLRNGICFPGICRRPYYWIGTCNNGIGSCCARGWR

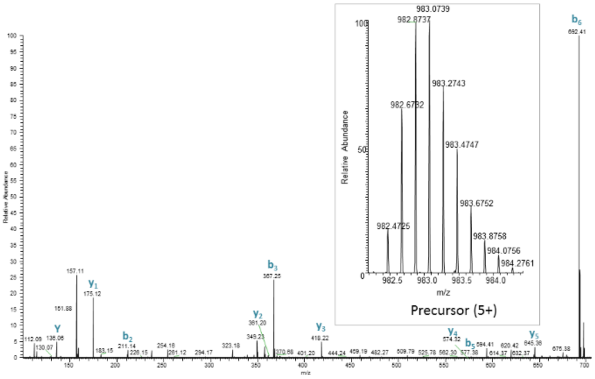

AvBD1 : full-length

GRKSDCFRKSGFCAFLKCPSLTLISGKCSRFYLCCKRIW-NH_2

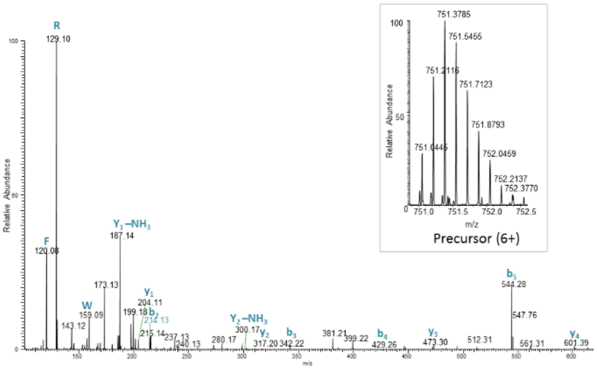

AvBD1 : peptidoform 1

KSDCFRKSGFCAFLKCPSLTLISGKCSRFYLCCKRIW-NH_2

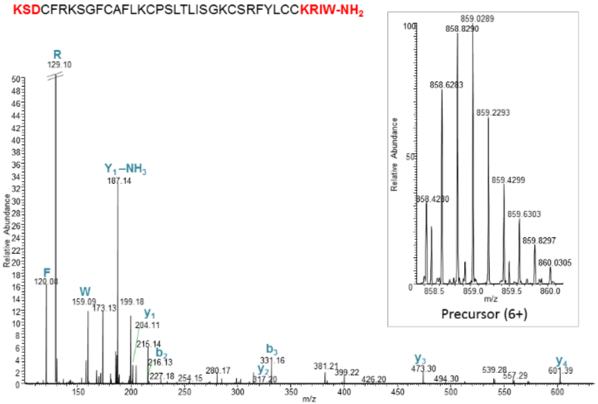

Supplement: S1 Fig — Interpreted nanoESI HR-MS and HR-MS/MS spectra are presented. In each HCD fragmentation spectrum obtained for (A) AvBD2 and (B) AvBD7 and AvBD1, a zoom of the multicharged precursor is presented with the annotated fragmentation spectra with the multicharged b- and y-ions. Identified sequences with the b- and y-type ions observed are indicated in red in the sequence. The three disulfide bridges are taken into account for all forms. C-terminal amidation and N-terminal pyroglutamic acid post-translation modifications are taken into account only for AvBD1 and AvBD7, respectively. Each table resume, for each AvBD, the average precursors selected for MS/MS, the charge of precursor (z), the observed monoisotopic mass [M+H]+, the theoretical monoisotopic mass [M+H]+, the delta mass (Da) between the observed and the theoretical mass, the E-value corresponding to the probability of identification by the ProSight software, the number of b and y ions observed and the total number of ions. (PDF) [file pone.0161573.s001.pdf]
