# Supplementary figures and images for: The Unusual Resistance of Avian Defensin AvBD7 to Proteolytic Enzymes Preserves Its Antibacterial Activity
Source: PLoS One. 2016 Aug 25;11(8):e0161573. doi: 10.1371/journal.pone.0161573 (PMC4999073; doi:10.1371/journal.pone.0161573)

**A**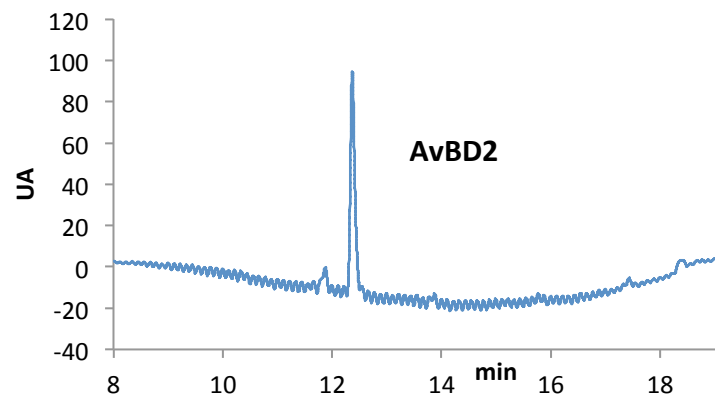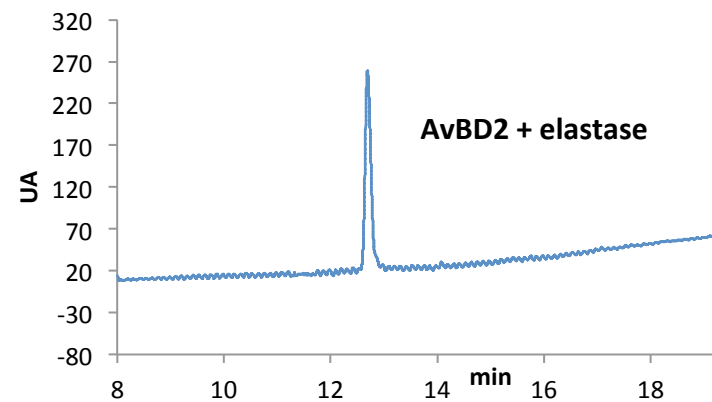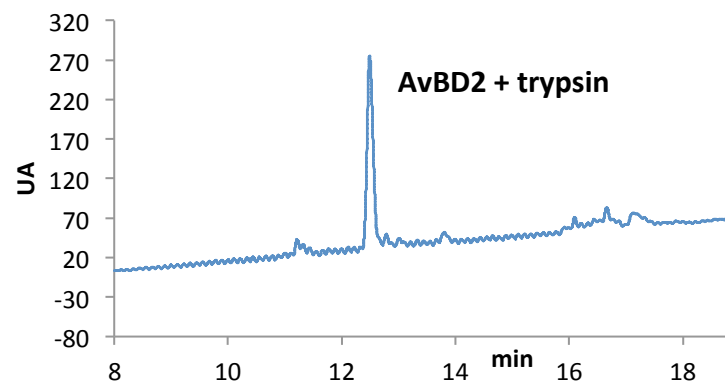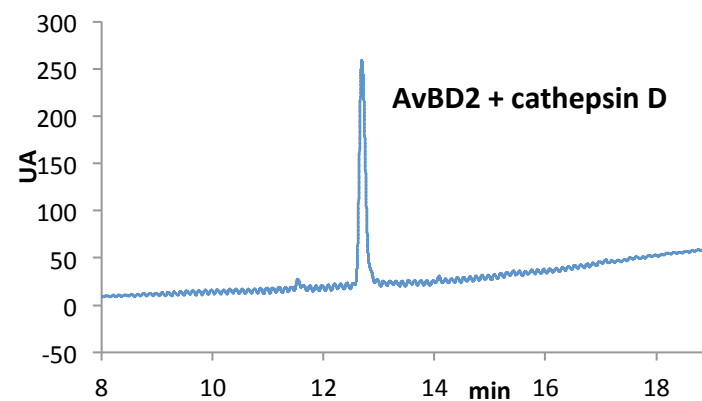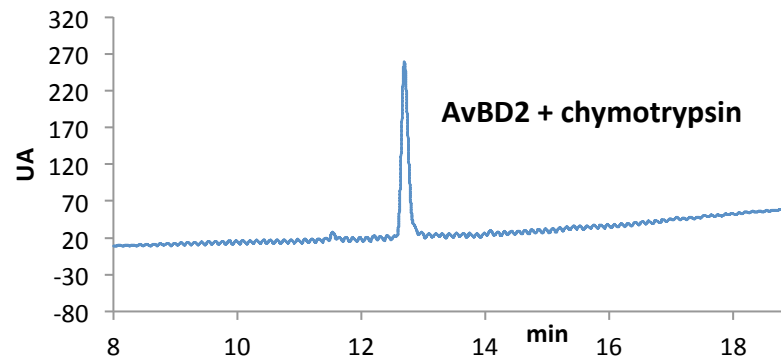

**B**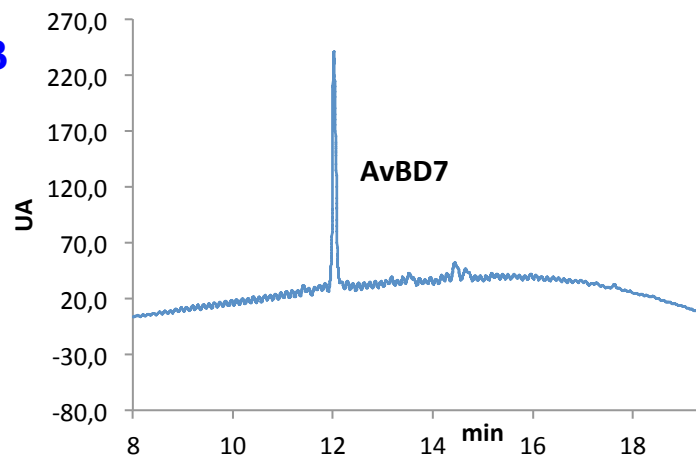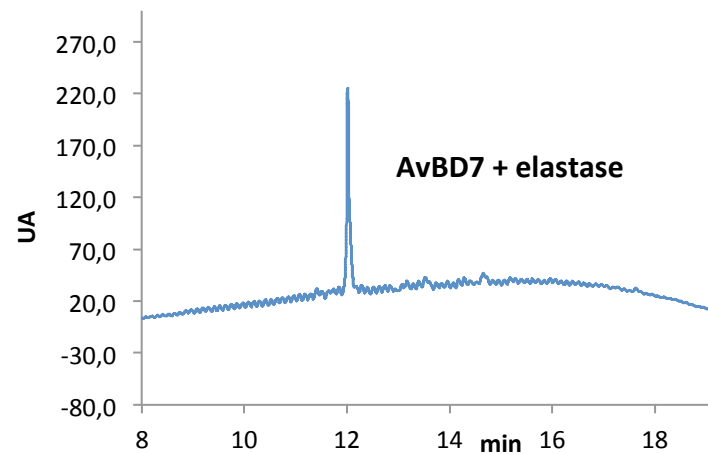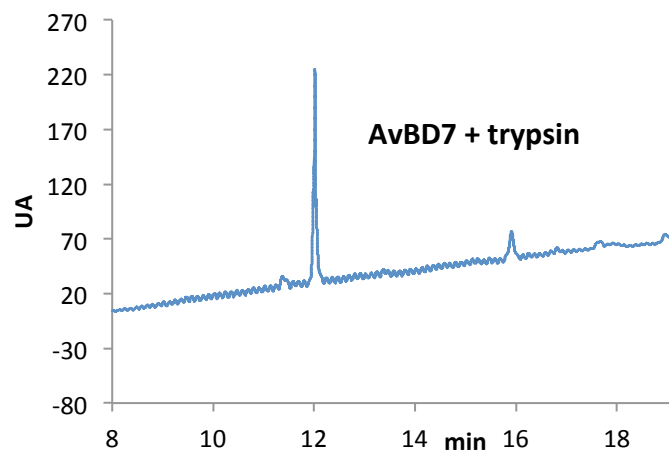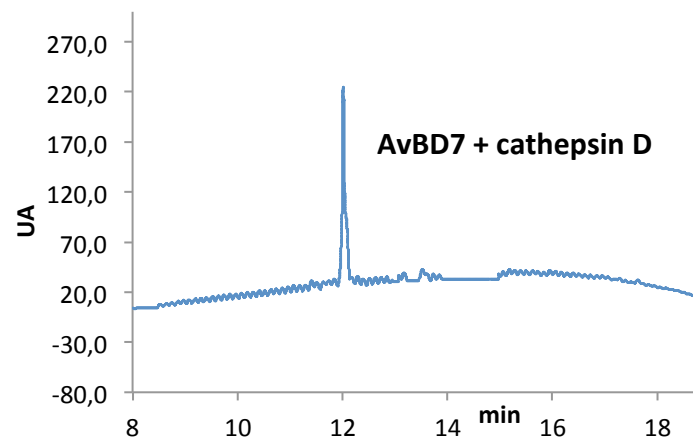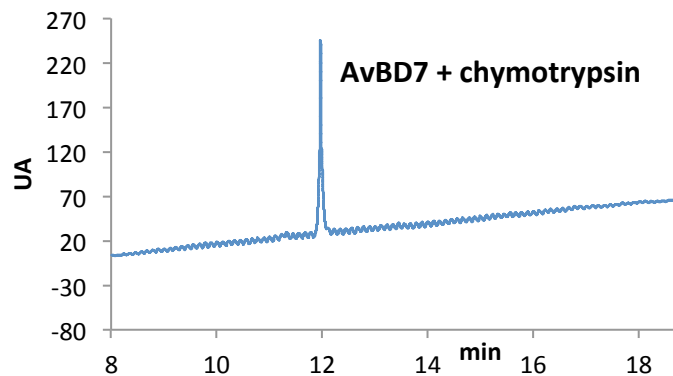

Supplement: S2 Fig — (A) AvBD2 (12.5 μM) was incubated in the presence of trypsin, chymotrypsin, neutrophil elastase or cathepsin D during 4 h at 30°C (substrate-enzyme ratio: 100) as described in detail in the experimental section. (B) The same procedure was repeated for AvBD7. Each reaction mixture was submitted to RP-HPLC (Brownlee ODS-032 column, 0–90% water/acetonitrile gradient in the presence of 0.1% TFA, flow rate of 1 mL/min; wavelength: 220 nm). Chromatograms were analyzed by running the ChromoQuest Chromatography Workstation (Thermo Fisher Scientific, Les Ulis, France). (PDF) [file pone.0161573.s002.pdf]
